# Effect of Shogaol on the Expression of Intestinal Stem Cell Markers in Experimentally Induced Colitis in BALB/c Mice

**DOI:** 10.1155/2019/5134156

**Published:** 2019-03-06

**Authors:** Snur M. A. Hassan, Ali Hussein Hassan

**Affiliations:** ^1^Department of Anatomy and Pathology, College of Veterinary Medicine, University of Sulaimani, Kurdistan, Iraq; ^2^Department of Medical Laboratory Sciences, Komar University of Science and Technology, Kurdistan Region, Iraq

## Abstract

**Aim:**

This study is aimed at investigating the effect of Shogaol, a phenolic constituent of ginger, on dextran sodium sulfate- (DSS-) induced ulcerative colitis (UC) in mice in comparison with 6-thioguanine (6-TG), an immune-suppressant chemotherapeutic medicine used for treatment of ulcerative colitis.

**Material & Methods:**

Thirty-six adult, male and female BALB/c mice were randomly divided into six groups: group 1 (control negative) not exposed to DSS and did not receive any treatment, group 2 (control positive) exposed to DSS but did not receive any treatment, group 3 exposed to DSS and treated by 0.1 mg/kg of 6-thioguanine, and groups 4, 5, and 6 exposed to DSS and treated by 10, 20, and 40 mg/kg b.w. Shogaol, respectively. At day 56, the mice were checked for their disease activity index (DAI) and they were sacrificed. The colons of the mice were examined for length measurement, histological index score, and the expression of CD133 and CD34 stem cell markers.

**Results:**

Shogaol showed a better curative effect than did 6-TG in repairing the colonic mucosal damages in DSS-exposed mice as indicated by the levels of CD133 and CD34 expression in the colonic crypts and by the DAI score, colon length measurements, & histological index score which were significantly reduced in mice treated by Shogaol, particularly the 20 and 40 mg/kg BW doses.

**Conclusion:**

The results of this study indicated that oral treatment with the ginger-derived substance Shogaol could be better than the conventional immunosuppressive chemotherapeutic remedy 6-TG in treatment of DSS-induced UC.

## 1. Introduction

Ulcerative colitis is a chronic inflammatory disease of the colon and rectum characterized by bloody diarrhea, intestinal mucosal ulceration, and infiltration of neutrophils and lymphocytes within the mucosal layer [1, 2]. Multiple causes such as environmental changes, gene variations, and gut microbes were supposed to be associated with UC etiology [3, 4]. DSS-induced colitis, a form of UC model in terms of morphological and pathophysiological features [5, 6], has been generally used experimentally to evaluate the therapeutic effects and to realize the molecular basis of action of new compounds to be used for the treatment of UC [5, 7].

Many of the therapeutic agents classically used for the treatment of UC, such as 6-thioguanine (also referred to as 6-TG or 2-amino-6-mercaptopurine), are immunosuppressants, and because UC commonly develops in elderly patients, there is a real risk of complications due to infection [8]. In addition, treatment with 6-TG is usually associated with unfavorable side effects such as hepatotoxicity, nephrotoxicity, and bone marrow suppression leading to anemia, leukopenia, and thrombocytopenia [9, 10].

Shogaol, a compound found in the rhizome of ginger (*Zingiber officinale* Roscoe) [11, 12], has been extensively reported for its numerous pharmacological properties including anti-inflammatory, analgesic, antipyretic, antioxidant, and anticancer properties [13, 14].

Properties that define potential stem cells (SC) include self-renewal, differentiation capacity, and asymmetric cell division via nonrandom chromosomal co-segregation [15]. These properties and several cluster differential (CD) membrane and cytoplasmic markers such as CD133, CD29, CD44, CD166 (ALCAM), EpCAM, ALDH1A1, and ALDH1B1 have been identified to investigate these properties and to isolate putative SC [16]. CD133, also known as PROML1 or prominin, was reported to show a characteristic expression pattern with localization toward the luminal surface of the colonic glands [17].

CD34 is a transmembrane sialomucin, and it has been extensively used as a marker of hematopoietic stem cells for nearly 30 years and more recently for other stem cell/progenitor types [18].

The present study is aimed at exploring the possible effect of Shogaol in repairing colonic damages in DSS-induced UC through activation of stem cells using CD133 and CD34 as markers for self-renewal and differentiation capacity of stem cells in the colonic crypts.

## 2. Material and Methods

### 2.1. Animals

Thirty-six male and female BALB/c mice (2-3 months of age) were purchased from the Animal House at the College of Veterinary Medicine, University of Sulaimani (Sulaymaniyah Governorate, Iraq). The mice were provided with tap water and standard food *ad libitum* and were allowed to acclimate for one week prior to the start of the experiment. Mice were housed at a density of 6/cage in air-conditioned housings with a room temperature of 24 ± 2°C, comparative humidity of 50 ± 10%, and interchanging 12-hour light/dark cycles. The experiment was accomplished throughout the light period of the cycle. All mouse-involving procedures in this study were carried out humanely and were performed according to the Guide for the Care and Use of Laboratory Animals and with the approval of the Ethics Committee at the College of Veterinary Medicine, University of Sulaimani.

### 2.2. Induction of Chronic Colitis and Shogaol Treatment

Ulcerative colitis was induced in all mice except those of the control negative group. Induction of UC was accomplished by exposing the mice to 3% DSS (molecular weight 40 kDa) via drinking water for four cycles (each cycle included 5 days of DSS exposure followed by a 7-day interval on normal water) [19]. Accordingly, the mice were allotted into 6 groups (6 mice per group) as follows: group 1 (control negative): the animals of which were left on normal water (no DSS exposure and no treatment); group 2 (control positive): DSS exposure with no treatment; group 3: DSS exposure followed by treatment with 0.1 mg/kg b.w. 6-thioguanine (Biochem Chemopharma, France) [20]; and groups 4, 5, and 6: DSS exposure followed by respective treatment with 10, 20, and 40 mg/kg b.w. (Shogaol, Sigma-Aldrich). All treatments (other than the 3% DSS exposure) were given as a single daily dose by oral gavages for 14 days (four treatment days with one treatment-free day interval).

### 2.3. Assessment of Colitis

Colitis was assessed in mice during the DSS exposure and Shogaol treatment, using a score of disease activity index (DAI, Table 1) depending on weight loss, stool consistency, and rectal bleeding [21].

After completion of the experiment period on day 56, the colon was resected and examined for its length, consistency of the stool, and gross appearance. The distal part of the colon was fixed immediately in 10% formalin, and a sequence of histopathological preparations was undergone according to Kiernan [22]. Tissue slices of 4 *μ*m thickness were attained and stained using the standard H and E technique, mounted on glass slides, visualized by light microscopy, and scored using a histological index (Table 2) to assess the ulcerative colitis severity [23]. All histological evaluations of the colon were achieved in a double-blinded manner by two professional pathologists.

### 2.4. Tissue Microarray Assembly

The most representative areas, on the H and E-stained slides, were carefully selected and labeled. The tissue microarrays (TMA) were collected using a tissue microarray instrument (TMA Builder, Thermo Scientific™) consisting of a thin-walled, stainless steel, punch needle, and stylet. The stylet, which closely fits the punch needle, is used to empty and transfer the needle content. The instrument was used to make holes in a recipient block with defined array cores arranged in 3 rows (4 cores per a row) in an X-Y position with a 2 mm increment between individual arrays and a 3 mm punch depth.

Four tissue sections, 4 *μ*m each, were obtained from each tissue microarray block for immunohistochemical staining using a rotary microtome (Leica). The sections were fixed on adhesive-coated slides, dewaxed in xylene at 55°C (3 times, 5 minutes each), and rehydrated by a series of washes in 100, 90, and 70% ethanol and distilled water (2 times, 5 minutes each). Antigen retrieval was performed by heating in a Dako pressure cooker for 20 minutes in 250 mL of 10 mmol/L sodium citrate (pH 6.0).

### 2.5. Immunohistochemical Staining

Endogenous peroxidase activity was blocked by dipping the slides in 0.3% hydrogen peroxidase for 15 minutes and washed by PBS (3 × 2 min), and then the sections were covered with 3% goat serum for about 1 hour to block nonspecific bindings. Following that, the slides were divided into 2 sets: the first set was incubated overnight at 4°C with rabbit anti-CD133 polyclonal Abs (1 : 100, San Francisco Biorbyt, USA), and the second set was incubated with rabbit anti-CD34 monoclonal Abs (1 : 100, Dako, Germany). The slides were then washed in PBS (3 × 2 min), incubated with a biotinylated goat anti-rabbit secondary antibody (Envision, Bio SB) for 30 minutes, and washed with PBS (3 × 2 min). Then after, The sections were incubated in a horseradish peroxidase-streptavidin (EnVision, Bio SB) for 30 minutes, washed with PBS (3 × 2 min), developed with DAB substrate for 10 minutes, counterstained with hematoxylin, dehydrated following a standard procedure, and covered with coverslips.

### 2.6. Immunofluorescent Staining

The tissue sections were permeabilized by covering them with few drops of (PBST 0.1% Triton) for 10 minutes. The sections were then washed in PBS (2 × 2 min) and incubated with a blocking solution containing 5% bovine serum in PBST for 30 minutes at room temperature. Following that, the sections were blotted and incubated overnight (in a dark jar at 4°C) with primary antibodies (polyclonal rabbit anti-CD133 antibody, San Francisco Biorbyt, USA), washed in PBS (3 × 2 min) in a dark jar, incubated with fluorescein isothiocyanate (FITC)-conjugated secondary antibody for 1 hour in a dark jar (San Francisco Biorbyt, USA), washed in PBS (3 × 5 min), covered with coverslips using a mounting medium, sealed with a nail polish, and stored in the dark at -20 or +4°C.

### 2.7. Interpretation of CD133 and CD34 Staining

Immunohistochemical staining of CD133 and CD34 among the colonic crypts was evaluated semiquantitatively by two independent pathologists in a blinded manner without knowledge of clinical and pathological information. The CD133 staining was considered positive in cases of cytoplasmic and/or membranous reactivity in epithelial cells of colonic crypts, and the CD34 staining was regarded positive in cases of membranous reactivity in the colonic cryptal epithelium and cytoplasmic reactivity in mesenchymal cells (macrophages, fibroblasts, and/or mast cells) in the lamina propria of the colonic mucosa.

The extent of positively stained epithelial cells in IHC staining of CD133 and CD34 was estimated by a five-point scale as no staining or 0 for <5%, 1 for 5-25%, 2 for 25-50%, 3 for 50-75%, and 4 for >75% positive staining. Staining intensity of CD133 and CD34 was graded into three levels: weak (+1), moderate (+2), and strong (+3). A total staining score was obtained by multiplying the positive reactivity extent and level of staining intensity making a range of 0-12 [24–26]. A positive immunofluorescence staining of CD133 was indicated by manifestation of a specific reaction represented by the appearance of a bright apple green fluorescence for the FITC-labeled secondary antibodies in the cytoplasm of the mucosal crypt epithelium. The intensity of immunoreactivity by immunofluorescence staining was categorized into three levels: weak (one +), moderate (two +), and strong (three +) [27].

### 2.8. Statistical Analysis

Statistical analysis was performed using SPSS version 22.0 software (SPSS, Chicago, IL, USA). The statistical analysis of variation among the experimental groups was performed by the one-way ANOVA test to examine DAI scores and the colon length. The results are presented as mean ± standard error (SE), and the values of *P* < 0.05 were regarded as statistically significant.

## 3. Results

### 3.1. Disease Activity Index (DAI) Score

**Figure 1:**
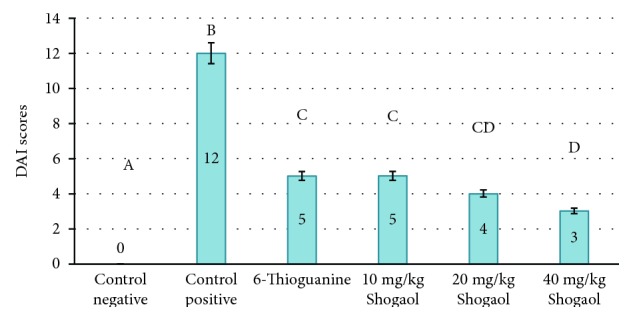
DAI scores in all experimental groups. The DAI scores are expressed as mean ± SE. DAI scores with different alphabetical letters superscript are significantly different (*P* < 0.05).

The results of the disease activity index (Figure 1) revealed a zero score for the control negative mice which showed no disease symptoms in comparison with the control positive mice “DSS exposure without treatment” which exhibited prominent blood in their stool, diarrhea, and rectal bleeding (score 12). On the other hand, the DAI scores of mice in group 3 (DSS exposure and 6-TG treatment), group 4 (DSS exposure and 10 mg/kg BW Shogaol treatment), group 5 (DSS exposure and 20 mg/kg BW Shogaol treatment), and group 6 (DSS exposure and 20 mg/kg BW Shogaol treatment) were 5, 5, 4, and 3, respectively.

### 3.2. Colon Length Measurements

**Figure 2:**
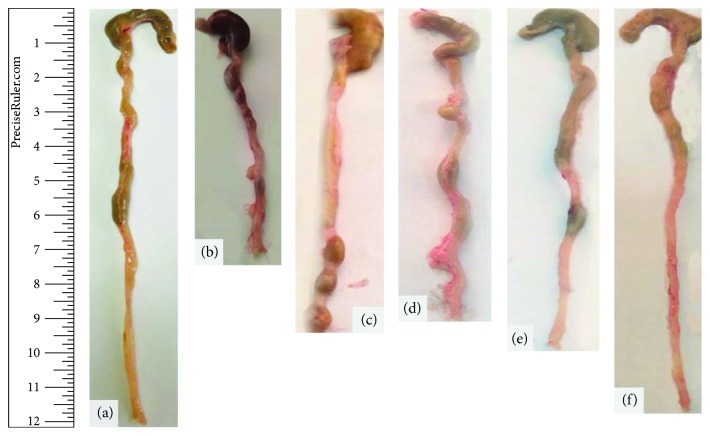
Representative colon images illustrated colon length as an indicator for ulcerative colitis in comparison with healthy colon of a control negative mouse. (a) Control –ve, (b) Control +ve (DSS exposure), (c) DSS exposure and 6-TG treatment, (d) DSS exposure and 10 mg/kg b.w. Shogaol treatment, (e) DSS exposure and 20 mg/kg b.w. Shogaol treatment, and (f) DSS exposure and 40 mg/kg b.w. Shogaol treatment.

In comparison with mice of the control negative group, a highly significant decrease (*P* < 0.001) was apparent in the average colon length in mice of the control positive group “DSS exposure without treatment” and a significant decrease (*P* < 0.05) in mice of the 6-thioguanine, 10 mg/kg BW, and 20 mg/kg BW Shogaol treatment groups. On the other hand, mice of the 40 mg/kg b.w. Shogaol treatment group showed only minimal nonsignificant decrease in the average colon length (Figure 2 and Table 3).

### 3.3. Histological Score Assessment of Chronic Colitis

**Figure 3:**
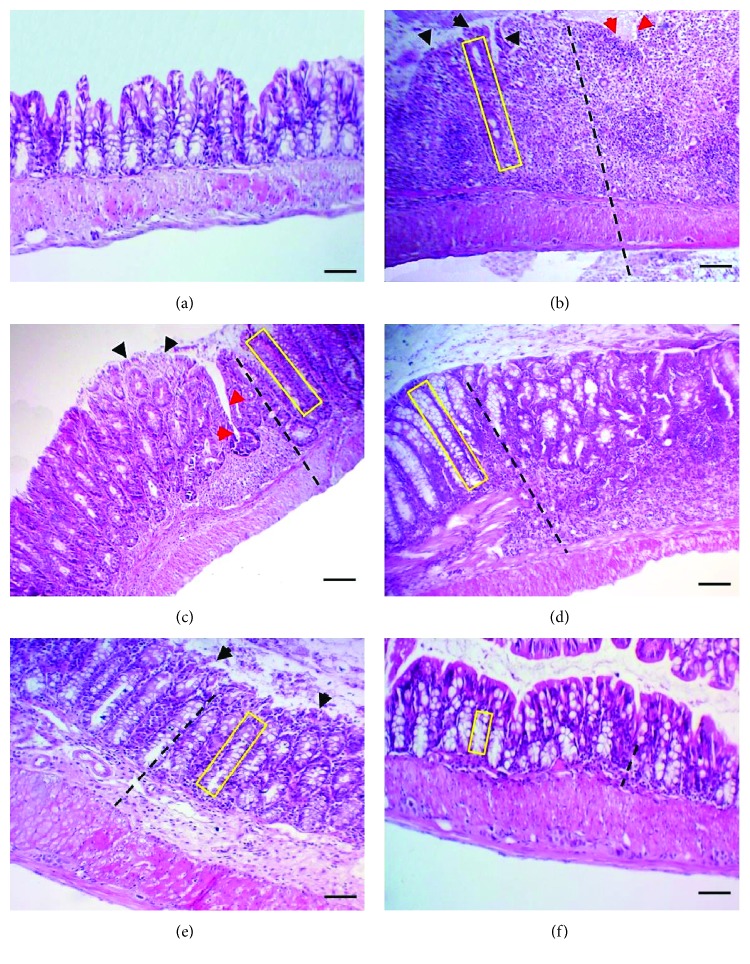
Microscopic view and total histological index score of the colon. (a) Normal colon appearance (sum score 0) in the control negative group. (b) Marked transmural infiltration of inflammatory cells, extensive epithelial erosion (black head arrows), focal ulceration (red head arrows), and marked epithelial hyperplasia + crypt loss (sum score 10) in the control positive group (DSS exposure without treatment). (c) Moderate inflammation in mucosa and submucosa, focal epithelial erosion (black head arrows), mild epithelial hyperplasia, and mild goblet cell loss (sum score 5) in group 3 (DSS exposure & 6-TG treatment). (d) Moderate-marked infiltration of inflammatory cells in the mucosa and submucosa, focal epithelial erosion (black head arrows), moderate epithelial hyperplasia, and moderate goblet cell loss (sum score 7) in group 4 (DSS exposure & 10 mg/kg b.w. Shogaol treatment). (e) Moderate infiltration of inflammatory cells in the mucosa and submucosa, focal epithelial erosion (black head arrow), mild epithelial hyperplasia, and mild goblet cell loss (sum score 5) in group 5 (DSS exposure & 20 mg/kg b.w. Shogaol treatment). (f) Mild infiltration of inflammatory cells in the mucosa and intact epithelium and no hyperplasia with no goblet cell loss (sum score 1) in group 6 (DSS exposure & 40 mg/kg b.w. Shogaol treatment). H&E stain. The black dashed line indicates the extent of inflammatory cells infiltration, and the yellow rectangle shows epithelial hyperplasia. Scale bar: 100 *μ*m.

In comparison with mice of the control negative group which shows normal colon morphology, different severity levels of colon inflammation were apparent in the different groups of DSS-exposed mice as indicated by infiltration of inflammatory cells, epithelial erosions or ulcerations, epithelial hyperplasia, goblet cell loss, and abnormal crypt morphology “crypt loss, irregularity, or abscesses” (Figure 3). The total histological index score ranges from zero, for the apparently normal colon of control negative mice, to 10, for the severely inflamed colon of the control positive mice (DSS exposure without treatment). The total histological index score of the colon in mice of the treatment groups was 5 in group 3 “DSS exposure with 6-TG treatment,” 7 in group 4 “DSS exposure with 10 mg/kg b.w. Shogaol treatment,” 5 in group 5 “DSS exposure with 20 mg/kg b.w. Shogaol treatment,” and only 1 in group 6 “DSS exposure with 40 mg/kg b.w. Shogaol treatment.”

### 3.4. CD133 and CD34 Expression

**Figure 4:**
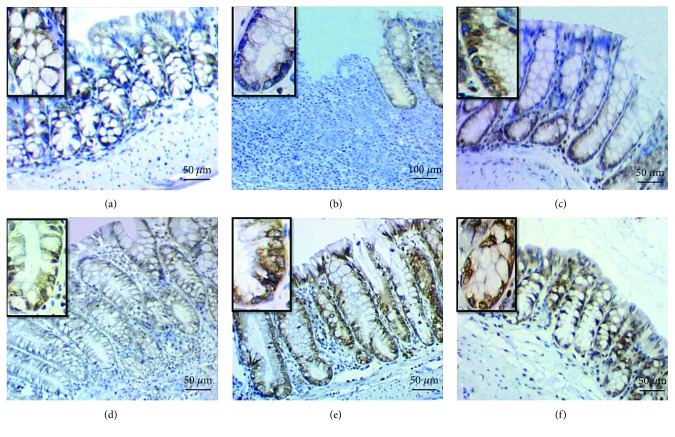
Cytoplasmic and membranous CD133 expression in epithelial cells of the colonic crypts. (a) Minimal, week expression (sum score 0) in mice of control negative group. (b) Focal, weak expression (sum score 1) in control positive group. (c) Focal, weak-moderate expression (sum score 4) deeply within the colonic crypt in group 3 (DSS exposure & 6-TG treatment). (d) Diffuse, weak expression (sum score 3) in group 4 (DSS exposure & 10 mg/kg b.w. Shogaol treatment). (e) and (f) Diffuse, moderate expression (sum score 6) extends into the transient amplifying and apical regions of the colonic crypts in group 5 (DSS exposure & 20 mg/kg b.w. Shogaol treatment) and group 6 (DSS exposure & 40 mg/kg b.w. Shogaol treatment), respectively.

Immunohistochemical staining of the colonic tissue sections revealed variable scores of cytoplasmic and membranous CD133 expression in epithelial cells of the mucosal crypts in different groups of mice (Figure 4). Minimal expression (sum score 0) was apparent in mice of group 1 (negative control); focal, weak expression (sum score 1) in group 2 (positive control “DSS exposure without treatment”); focal, weak-moderate expression (sum score 4) deeply within the colonic crypt in group 3 (DSS exposure and 6-TG treatment); weak but diffuse expression (sum score 3) in group 4 (DSS exposure and 10 mg/kg b.w. Shogaol treatment); and moderate and diffuse expression (sum score 6) not limited to the crypt niche but also extended into the transient amplifying region and the differentiated colonocytes in the apical portion of the colonic crypts in group 5 (DSS exposure and 20 mg/kg b.w. Shogaol treatment) and group 6 (DSS exposure and 40 mg/kg b.w. Shogaol treatment).

**Figure 5:**
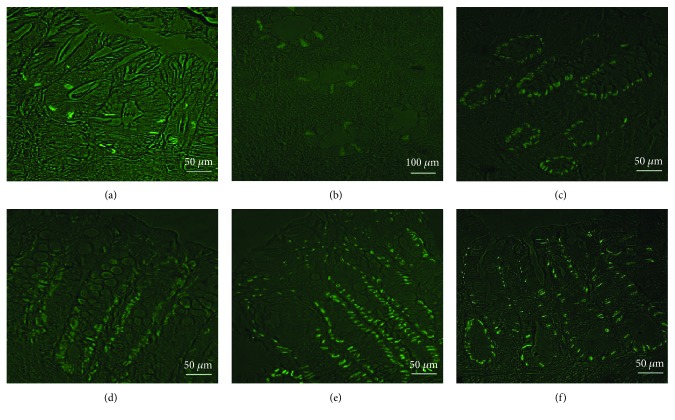
Immunofluorescence expression of the CD133 protein in the cytoplasm of the colonic cryptal epithelium. (a) and (b) Weak (+) expression in mice of the control negative and control positive group. (c) and (d) Weak-moderate (+ to ++) expression in the in deep portions of the colonic crypts in mice of group 3 (6-TG treatment) and group 4 (10 mg/kg b.w. Shogaol treatment). (e) and (f) Moderate (++) expression in mice of group 5 (20 mg/kg b.w. Shogaol treatment) and mice of group 6 (40 mg/kg b.w. Shogaol treatment).

Similarly, the immunofluorescence staining of the colonic tissue sections revealed variable scores of cytoplasmic CD133 expression in epithelial cells of the mucosal crypts in different groups of mice (Figure 5). Weak expression (+) was apparent in mice of group 1 (negative control) and group 2 (positive control “DSS exposure without treatment”), weak-moderate expression (+ to ++) in deep portions of the colonic crypts in mice of group 3 (DSS exposure & 6-TG treatment) and group 4 (DSS exposure and 10 mg/kg b.w. Shogaol treatment), and moderate expression (++) in mice of group 5 (DSS exposure and 20 mg/kg b.w. Shogaol treatment) and group 6 (DSS exposure and 40 mg/kg b.w. Shogaol treatment).

**Figure 6:**
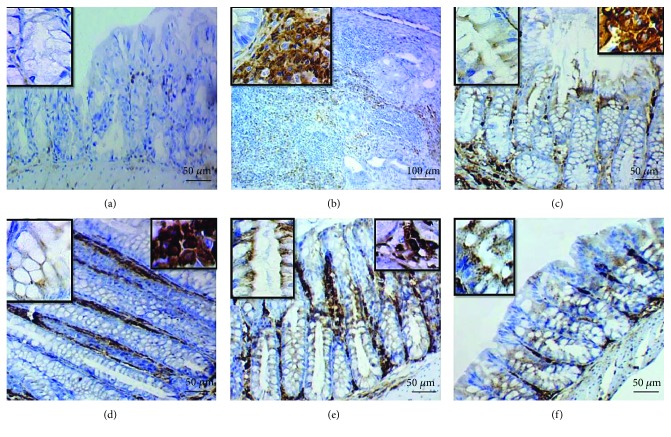
Membranous CD34 expression the mucosal cryptal epithelium and cytoplasmic expression in macrophages, fibroblasts, and mast cells in the lamina propria of the colon. (a) Negative expression (sum score 0) in mice of the control negative group. (b) Negative membranous expression in the mucosal cryptal epithelium and moderate cytoplasmic expression in macrophages, fibroblasts, and mast cells of colonic lamina propria (sum score 1) in the control positive group. (c) Weak-moderate expression in both mucosal crypts and lamina propria (sum score 3) in mice of group 3 (DSS exposure & 6-TG treatment). (d) Weak expression in both mucosal crypts and lamina propria (sum score 2) in mice of group 4 (DSS exposure &10 mg/kg b.w. Shogaol treatment). (e) and (f) Moderate-strong expression in both mucosal crypts and lamina propria (sum score 6) in mice of group 5 (DSS exposure & 20 mg/kg b.w. Shogaol treatment) and group 6 (DSS exposure and 40 mg/kg b.w. Shogaol treatment).

In relation to IHC staining of CD34, variable scores of membranous expressions were seen in epithelial cells of the mucosal crypts and cytoplasmic expression in mesenchymal cells (macrophages, fibroblasts, and/or mast cells) in the lamina propria of the colonic mucosa (Figure 6). No expression was apparent in both mucosal crypts and mesenchymal cells of the colon (sum score 0) in control negative mice; negative membranous expression in the mucosal cryptal epithelium and weak cytoplasmic expression in mesenchymal cells of the colonic mucosa (sum score 1) in control positive mice (DSS exposure without treatment); weak-moderate expression in mucosal crypts and mesenchymal cells (sum score 3) in mice of group 3 (DSS exposure and 6-TG treatment); minimal, weak expression in mucosal crypts and mesenchymal cells (sum score 2) in mice of group 4 (DSS exposure + Shogaol treatment); and moderate expression in mucosal crypts and mesenchymal cells (sum score 6) in mice of group 5 (DSS exposure and 20 mg/kg b.w. Shogaol treatment) and group 6 (DSS exposure and 40 mg/kg b.w. Shogaol treatment).

## 4. Discussion

As an inflammatory bowel disease (IBD), ulcerative colitis is regarded as an important and increasing health care crisis worldwide, and the precise etiology remains unknown [28]. However, intestinal regeneration plays an important role in the healing of the intestinal mucosa [29]. In comparison with mice of the control negative group, the control positive mice showed severe signs of ulcerative colitis (as indicated by DAI parameters including bloody stool, diarrhea, and rectal bleeding) associated with colon shortening and marked histopathological lesions in the colon represented by mucosal damages (focal erosion or ulceration) and mucosal-submucosal or transmural infiltration of inflammatory cells. These results are in agreement with some related findings [30, 31] which reported that DSS-induced UC in the murine model causes adverse colonic abnormalities associated with bloody stool, diarrhea, and rectal bleeding.

On the other hand, the signs and features of DSS-induced UC are shown to be reduced in mice of group 3 (DSS exposure and 6-TG treatment) and in mice of groups 4, 5, and 6 (DSS exposure followed by oral doses of 10, 20, and 40 mg/kg BW shogaol, respectively) as indicated by the DAI score and colon length measurements which were lower than those of the mice in the control positive group. These results are compatible with the findings of another related work, achieved by the authors [32], dealt with using the Shogaol for treatment of acute ulcerative colitis and showed that this compound has protected the mice against body weight loss caused by DSS-induced colitis and has resulted in a significant reduction in DAI score due to its anti-inflammatory effect as indicated by the decrease in expression of the epidermal growth factor receptor in the colonic tissue sections.

Interestingly, the histological index score values of group 6 mice (DSS exposure followed by oral doses of 40 mg/kg BW shogaol) were lower than the comparable values of group 3 mice (DSS exposure and 6-TG treatment). In addition, mice of group 6 showed only minimal infiltration of inflammatory cells in the mucosal and submucosal layers of the colon with no epithelial damage or hyperplasia whereas mice of group 3 showed moderate changes in colonic sections such as moderate inflammation in the mucosa and submucosa, focal epithelial erosion with mild epithelial hyperplasia, and goblet cell loss. These results indicate that the 40 mg/kg b.w. dose of Shogaol could be better than 6-TG in the treatment of UC and they are generally compatible with the findings of Zhang et al. [33], who stated that oral delivery of nanoparticles loaded with 6-Shogaol is able to attenuate inflammation of the colon in a murine model of UC.

The results of IHC staining of colonic tissue sections revealed variable degrees of CD133 expression in the different groups of mice that were exposed to DSS in comparison with the control negative group which showed a negative expression. A focal, weak expression was apparent in the control positive group; focal, weak-moderate expression in group 3 (DSS exposure & 6-TG treatment); diffuse, weak expression in group 4 (DSS exposure & 10 mg/kg b.w. Shogaol treatment); and diffused, moderate expression in group 5 (DSS exposure & 20 mg/kg b.w. Shogaol treatment) and group 6 (DSS exposure & 40 mg/kg b.w. Shogaol treatment). These findings revealed that the different types of treatments performed in this study have resulted in variable improvement levels of DSS-induced colitis. The CD133 expression in the 20 and 40 mg/kg b.w. Shogaol treatment groups was not limited to the crypt niche but also extended into the transient amplifying region and into the differentiated colonocytes in the apical portion of the colonic crypts in comparison with the focal, weak-moderate expression deeply within the colonic crypts in the 6-TG treatment group, indicating that Shogaol is possibly better than 6-TG in the treatment of UC. This result is in agreement with the findings of Karim et al. [34] who stated that CD133 has a significant role in intestinal regeneration, and its signaling plays an important role in regulating intestinal homeostasis.

Variable scores of CD34 expression were also seen in epithelial cells of the mucosal crypts and in mesenchymal cells of the colonic mucosa in the different groups of DSS-exposed mice compared to the control negative mice which exhibited a negative expression. No expression was apparent in the mucosal cryptal epithelium and moderate expression in mesenchymal cells in mice of control positive mice (DSS exposure without treatment); a weak-moderate expression in both mucosal crypts and mesenchymal cells was apparent in group 3 (DSS exposure and 6-TG treatment); a minimal, weak expression in both mucosal crypts and mesenchymal cells was apparent in group 4; and a moderate-strong expression in both mucosal crypts and mesenchymal cells was apparent in group 5 (DSS exposure & 20 mg/kg BW Shogaol treatment) and group 6 (DSS exposure and 40 mg/kg BW Shogaol treatment). These results indicated a better intestinal regeneration in DSS-induced UC in mice treated with 20 and 40 mg/kg BW Shogaol than in mice treated by the conventional immunosuppressive chemotherapeutic remedy 6-TG. This finding is compatible with that of Pull et al., who reported that activation of macrophages represents an adaptive response of the colonic epithelial progenitor niche to injury [35], and it is also in accordance with the finding of Stzepourginski *et al*., who specified that CD34+ is an important factor in the IESC niche at homeostasis and it contributes to intestinal inflammation and repair after damage [36].

## 5. Conclusion

The results of the current study indicated that oral treatment by the ginger-derived substance Shogaol, particularly the 20 and 40 mg/kg b.w. doses, could be better than the conventional immunosuppressive chemotherapeutic remedy 6-TG in the treatment of DSS-induced UC because treatment by the latter compound is usually associated with unfavorable side effects such as hepatotoxicity, nephrotoxicity, and bone marrow suppression. The DAI parameters (bloody stool, diarrhea, and rectal bleeding), colon measurements, and histopathological index scores revealed a better positive effect of Shogaol in improvement of colitis due to its anti-inflammatory effect than 6-TG, and the IHC analysis also revealed a better effect for Shogaol than 6-TG in activation of CD133 which has a significant role in intestinal regeneration and in regulating intestinal homeostasis. In addition, the IHC results showed a better effective role of Shogaol than 6-TG in the activation of CD34 which plays an important role in improving crypt damages through the activation of mesenchymal cells which represent a major component of the intestinal stem cell niche in homeostasis and after injury.

## Figures and Tables

**Table 1:** Disease activity index score (DAI score).

Score	Weight loss	Stool consistency	Rectal bleeding
0	None	Normal	No bleeding
1	1-5%	—	—
2	5-10%	Loose stool	Mild bleeding
3	10-15%	—	—
4	More than 15%	Watery diarrhea	Prominent bleeding

Sum of scores: a range of 0-12.

**Table 2:** Histological scoring scheme for chemically induced colonic inflammation in chronic model [23].

Inflammatory cell infiltration (score 1)			Intestinal architecture (score 2)		
Severity	Extent	Score value	Epithelial changes	Mucosal architecture	Score value
Normal	Normal cellularity	0	Intact or normal	Normal	0
Minimal	Mucosa	1	Minimal hyperplasia	—	1
Mild	Mucosa, sometimes extending into submucosa	2	Mild hyperplasia, minimal goblet cell loss ± erosions		2
Moderate	Mucosa and submucosa	3	Moderate hyperplasia ± moderate goblet cell loss ± erosions	± Focal ulcerations	3
Marked	Mucosa and submucosa	4	Marked hyperplasia	± Irregular crypts or crypt loss ± ulcerations	4
Marked	Transmural	5	Marked hyperplasia ± multiple crypt abscesses	± Irregular crypts or crypt loss ± ulcerations	5

Sum of scores 1 and 2: a range of 0-10.

**Table 3:** Colon length measurements in different mice groups of the study.

Groups	Average colon length ± SE (cm)
Group 1 (control negative)	12.33 ± 0.24^a^
Group 2 (control positive “DSS exposure without treatment”)	7.70 ± 0.24^b^
Group 3 (DSS exposure + 6-TG treatment)	9.32 ± 0.10^c^
Group 4 (DSS exposure + 10 mg/kg BW Shogaol treatment)	9.13 ± 0.20^c^
Group 5 (DSS exposure + 20 mg/kg BW Shogaol treatment)	10.05 ± 0.18^c^
Group 6 (DSS exposure + 40 mg/kg BW Shogaol treatment)	11.56 ± 0.18^a^

The average colon lengths are expressed by mean ± standard error. Average colon length values with different alphabetical letter superscripts are significantly different. ^b^(*P* < 0.01) and ^c^(*P* < 0.05).

## Data Availability

All data used to support the findings of this study are included within the article.
